# Usefulness of the prostate health index in predicting the presence and aggressiveness of prostate cancer among Korean men: a prospective observational study

**DOI:** 10.1186/s12894-021-00897-2

**Published:** 2021-09-16

**Authors:** Jae Yoon Kim, Ji Hyeong Yu, Luck Hee Sung, Dae Yeon Cho, Hyun-Jung Kim, Soo Jin Yoo

**Affiliations:** 1 Department of Urology, Sanggye Paik Hospital, Inje University College of Medicine, 1342 Dongil-ro, Nowon-gu, Seoul, 01757 Republic of Korea; 2 Department of Pathology, Sanggye Paik Hospital, Inje University College of Medicine, 1342 Dongil-ro, Nowon-gu, Seoul, 01757 Republic of Korea; 3 Department of Laboratory Medicine, Sanggye Paik Hospital, Inje University College of Medicine, 1342 Dongil-ro, Nowon-gu, Seoul, 01757 Republic of Korea

**Keywords:** Prostate health index, Prostate-specific antigen, Prostate cancer, Korean

## Abstract

**Background:**

We aimed to evaluate the usefulness of the Beckman Coulter prostate health index (PHI) and to compare it with total prostate-specific antigen (PSA) levels and related derivatives in predicting the presence and aggressiveness of prostate cancer (PCa) in the Korean population.

**Methods:**

A total of 140 men who underwent their first prostate biopsy for suspected PCa were included in this prospective observational study. The diagnostic performance of total PSA, free PSA, %free PSA, [–2] proPSA (p2PSA), %p2PSA, and PHI in detecting and predicting the aggressiveness of PCa was estimated using the receiver operating characteristic curve (ROC) and logistic multivariate regression analyses.

**Results:**

Of 140 patients, PCa was detected in 63 (45%) of participants, and 48 (76.2%) of them had significant cancer with a Gleason score (GS) ≥ 7. In the whole group, the area under the curve (AUC) for ROC analysis of tPSA, free PSA, %fPSA, p2PSA, %p2PSA, and PHI were 0.63, 0.57, 0.69, 0.69, 0.72, and 0.76, respectively, and the AUC was significantly greater in the PHI group than in the tPSA group (*p* = 0.005). For PCa with GS ≥ 7, the AUCs for tPSA, free PSA, %fPSA, p2PSA, %p2PSA, and PHI were 0.62, 0.58, 0.41, 0.79, 0.86, and 0.87, respectively, and the AUC was significantly greater in the PHI group than in the tPSA group (*p* < 0.001). In the subgroup with tPSA 4–10 ng/mL, both %p2PSA and PHI were strong independent predictors for PCa (*p* = 0.007, *p* = 0.006) and significantly improved the predictive accuracy of a base multivariable model, including age, tPSA, fPSA and %fPSA, using multivariate logistic regression analysis. (*p* = 0.054, *p* = 0.048). Additionally, at a cutoff PHI value > 33.4, 22.9% (32/140) of biopsies could be avoided without missing any cases of aggressive cancer.

**Conclusions:**

This study shows that %p2PSA and PHI are superior to total PSA and %fPSA in predicting the presence and aggressiveness (GS ≥ 7) of PCa among Korean men. Using PHI, a significant proportion of unnecessary biopsies can be avoided.

**Supplementary Information:**

The online version contains supplementary material available at 10.1186/s12894-021-00897-2.

## Background

Prostate cancer (PCa) is the second most common cancer in the Western population [1].

In Korea, the incidence of PCa has steadily increased, and it is now the fifth most common cancer among men [2]. Several studies have demonstrated that PCa in Korean men shows worse disease characteristics [3]. Kang and colleagues demonstrated that Koreans had higher T stages compared to their American counterparts (*p* = 0.021) and higher Gleason scores compared to Americans in all age groups. Moreover, Koreans also had higher Gleason scores compared to Americans for PSA > 10 ng/mL (*p* < 0.05) in their study.

A large proportion of PCa cases diagnosed in the Korean population show poor differentiation compared to their American counterparts [4]. Therefore, the accuracy of diagnosis and risk stratification of PCa using appropriate biomarkers may be more important for suitable treatment in the Korean population.

Total prostate-specific antigen (tPSA) is a widely used tumour marker for the screening, diagnosis, monitoring, and prognosis of PCa worldwide [5]. The introduction of tPSA has resulted in increased early detection of PCa and reduced mortality [6]. In the early diagnosis of PCa using tPSA, the main problem was that the low positive predictive value (PPV) of tPSA resulted in unnecessary biopsies [7]. The positive rate for cancer at biopsy was approximately 25% among the population with PSA levels of 2–10 µg/L.

In addition, PSA cannot accurately identify aggressive PCa, which has clinical significance for treatment. Consequently, the wide application of PSA in detecting PCa has increased concerns about over-diagnosis and over-treatment [8].

Therefore, the development of new biomarkers is needed to improve the detection of PCa and to discriminate clinically significant and insignificant PCa.

Several studies have performed PSA isoform assays to overcome the limitations of PSA. Free PSA (fPSA) consists of three different forms: benign PSA, intact inactive PSA, and proPSA. The subfraction of proPSA has several molecular isoforms, [–2], [–4], and [–5, − 7] proPSA [9].

Previous studies have demonstrated a significantly increased detection rate for PCa by measuring [–2] proPSA (p2PSA), especially the derivatives %p2PSA (p2PSA/fPSA) and prostate health index (PHI), which is a mathematical combination of tPSA, fPSA, and p2PSA [10, 11]. Additionally, recent studies demonstrated that %p2PSA and PHI showed superior performance to tPSA or %fPSA (fPSA/tPSA) in predicting PCa aggressiveness [12].

The aim of this prospective, observational study was to investigate the usefulness of p2PSA and its derived %p2PSA and PHI in the detection of PCa and to discriminate clinically significant PCa in Korean patients by estimating its ability to avoid unnecessary biopsies.

## Methods

The study included consecutive men who underwent their first prostate biopsy between April 2016 and July 2019. The indications for prostate biopsies were any one of the following: serum tPSA > 3.0 ng/mL, presence of a palpable nodule on prostate digital rectal examination, and observation of hypoechoic findings on transrectal prostate ultrasonography. Exclusion criteria were medical therapies or procedures that might affect PSA levels, acute prostate inflammation, or urinary tract infection in the 3 months prior to biopsy. Patients who underwent prostate biopsy or were treated with any 5-alpha-reductase inhibitors were excluded.

Blood samples were collected to measure the pre-biopsy tPSA, fPSA, and p2PSA levels prior to prostate biopsy. The blood was processed to clot for 1 h at room temperature, followed by centrifugation for 15 min. The sera were aliquoted and frozen at -80° C and processed on an Access 2 immunoassay system (Beckman Coulter, Brea, CA, USA) using dedicated Access tPSA, fPSA, and p2PSA reagents. %p2PSA was calculated using the formula [(p2PSA pg/mL)/ (fPSA ng/mL × 1000)] × 100, and PHI using the formula [(p2PSA pg/ mL)/(fPSA ng/mL] × √tPSA.

The patients then underwent transrectal ultrasound-guided prostate biopsies following a standardised extended scheme with at least 12 biopsy cores obtained from the prostate gland and additional cores taken when other areas were suspected. The specimens were inspected by a genitourinary pathologist who was blinded to the results of the blood test. PCa was confirmed and graded according to the definitions of the International Society of Urological Pathology.

The primary endpoint was comparison of accuracy of the diagnostic performance of %p2PSA and PHI with that of tPSA and %fPSA, which are currently widely used biomarkers in detecting PCa at biopsy. The secondary endpoint was the predictive ability of these biomarkers to discriminate aggressive PCa with a Gleason score (GS) ≥ 7.

Quantitative data are presented as median (interquartile range) and categorical data as numbers (n) and percentages. The normal distribution of variables was assessed using the Kolmogorov-Smirnov test. Student’s t-test was used for comparisons of parametric variables, and the Mann-Whitney U-test for non-parametric continuous variables. Bivariate and multivariate logistic regression analyses were used to determine the association between the biomarkers and the presence of PCa in the whole group and subgroup with PSA 4–10 and PCa with GS ≥ 7 at biopsy.

These markers were added to the base multivariate model, including age, tPSA, fPSA and %fPSA, to evaluate the usefulness of %p2PSA and PHI in predicting the presence of PCa. The improvement in predictive accuracy was measured as the area under the curve (AUC) of the receiver operating characteristic (ROC) analysis. The DeLong method was used to compare the statistical differences between the AUCs. Odds ratios (ORs) with 95% confidence intervals were determined.

Statistical analyses were conducted using SPSS Statistics version 26 (IBM Corp., Armonk, NY, USA). Statistical significance was set at *p* < 0.05. AUC comparisons were conducted using MedCalc software 19.4 (MedCalc Software, Mariakerke, Belgium).

The study protocol was approved by the Institutional Review Board (IRB) of the Sanngye Paik Hospital, Inje University. All participants provided written informed consent before participation in the study. All methods were carried out in accordance with relevant guidelines and regulations.

## Results

A total of 140 men who underwent their first prostate biopsy with positive or negative prostate biopsy between April 2016 and July 2019 were included in this study. The demographic and clinical characteristics of the study participants are summarised in Table 1. The range of tPSA levels was 0.82–23.9 ng/mL for those without cancer and 1.63–91.7 ng/mL for those with cancer. Among all patients, 77 men had a tPSA level between 4 and 10 ng/mL. PCa at the initial biopsy was detected in 45% (63/140) of the patients. Among 63 patients diagnosed with PCa, 15 (23.8%) had GS 6 disease, 18 (28.6%) had GS 7 disease, and 30 (47.6%) had GS ≥ 8 disease.

Patients with PCa showed significantly higher age, tPSA, %p2PSA, and PHI compared to those without PCa at biopsy. Conversely, %fPSA levels were significantly higher in patients without PCa. However, the fPSA concentration did not differ between the two groups.

**Table 1:** Demographic and clinical characteristics of all study subjects who underwent the first prostate biopsy

	Total	No cancer	Cancer	*P* value
	N, 140	N, 77 (55%)	N, 63 (45%)	–
Age, yr	69.0 (10.0)	67.0 (9.0)	72.0 (10.0)	***0.001***
tPSA, ng/mL	6.93 (6.05)	6.45 (3.8)	8.18 (15.3)	***0.010***
fPSA	1.03 (0.92)	0.92 (0.8)	1.07 (1.4)	*0.160*
%fPSA	14.55 (9.29)	17.13 (8.0)	11.16 (9.0)	***0.003***
p2PSA, pg/mL	19.18 (30.97)	15.54 (16.7)	31.06 (58.6)	***< 0.001***
%p2PSA	2.02 (2.21)	1.57 (1.5)	2.83 (2.6)	***< 0.001***
PHI	45.90 (82.72)	39.54 (38.0)	91.18 (147.9)	***< 0.001***
Positive core number	–	–	5.0 (7.0)	–
*Number *(*percentage*)				
GS 6%	–	–	15 (23.8%)	–
GS 7			18 (28.6%)	–
GS ≥ 8			30 (47.6%)	–

Data are shown as median (interquartile range), or number (%)

PSA, prostate-specific antigen; tPSA, total PSA; fPSA, free PSA; p2PSA, [2]proPSA, PHI, prostate health index; GS, Gleason score

P value to be statiscally significant with bolditalics

In 77 patients with a tPSA level between 4 and 10 ng/mL, %p2PSA and PHI were significantly different between groups with and without PCa (Table 2). However, the median tPSA, %fPSA, and p2PSA levels did not differ between the two groups.

**Table 2:** Descriptive characteristics of subjects with tPSA 4–10 ng/mL

	Total	No cancer	Cancer	*P value*
	N, 77	N, 48 (62.3%)	N, 29 (37.7%)	–
Age, yr	68.0 (12.0)	66.5 (11.0)	71.0 (12.0)	***0.010***
tPSA, ng/mL	6.41 (2.5)	6.42 (2.3)	6.13 (2.8)	*0.877*
fPSA	0.92 (0.5)	0.97 (0.6)	0.92 (2.4)	*0.185*
%fPSA	15.49 (8.6)	16.83 (7.5)	13.04 (7.1)	*0.284*
p2PSA, pg/mL	16.86 (17.5)	15.34 (16.1)	17.09 (23.7)	*0.189*
%p2PSA	1.61 ( 1.6)	1.50 (1.0)	2.35 (2.6)	***0.015***
PHI	41.35 (37.9)	39.65 (28.9)	53.72 (56.8)	***0.018***
Positive core number	–	–	4.0 (7.0)	–
*Number (percentage)*				
GS 6	–	–	9 (31%)	–
GS 7			10 (34.5%)	–
GS ≥ 8			10 (34.5%)	–

Data are shown as median (interquartile range), or number (%)

PSA, prostate-specific antigen; tPSA, total PSA; fPSA, free PSA; p2PSA, [2]proPSA; PHI, prostate health index; GS, Gleason score

P value to be statiscally significant with bolditalics

In the PCa group, 48 (76.2%) patients with GS ≥ 7 showed significantly higher %p2PSA (3.17% vs. 1.26%, *p* <  0.001) and PHI (120.8 vs. 35.4, *p* <  0.001) compared to those with GS 6 disease (Table 3). Patients with GS ≥ 7 had a more positive core number (6.0 vs. 3.0, *p* = 0.032)

**Table 3:** Comparison between GS 6 disease and more aggressive disease in PCa patients

	GS 6	GS ≥ 7	*P* value
	N, 15 (23.8%)	N, 48 (76.2%)	–
Age, yr	73.0 (9.0)	72.0 (12.0)	*0.520*
tPSA, ng/mL	7.47 (4.6)	9.75 (23.6)	*0.156*
fPSA	0.97 (0.6)	1.13 (1.7)	*0.358*
%fPSA	13.08 (8.0)	10.98 (9.2)	*0.302*
p2PSA, pg/mL	13.9 (13.4)	45.3 (72.6)	***0.001***
%p2PSA	1.26 (1.0)	3.17 (2.2)	***< 0.001***
PHI	35.4 (28.0)	120.8 (148.6)	***< 0.001***
Positive core number	3.0 (4.0)	6.0 (6.0)	***0.032***

Data are shown as median (interquartile range), or number (%)

PSA , prostate-specific antigen; tPSA, total PSA; fPSA = free PSA; p2PSA, [2]proPSA; PHI, prostate health index; GS, Gleason score

P value to be statiscally significant with bolditalics

**Fig. 1:**
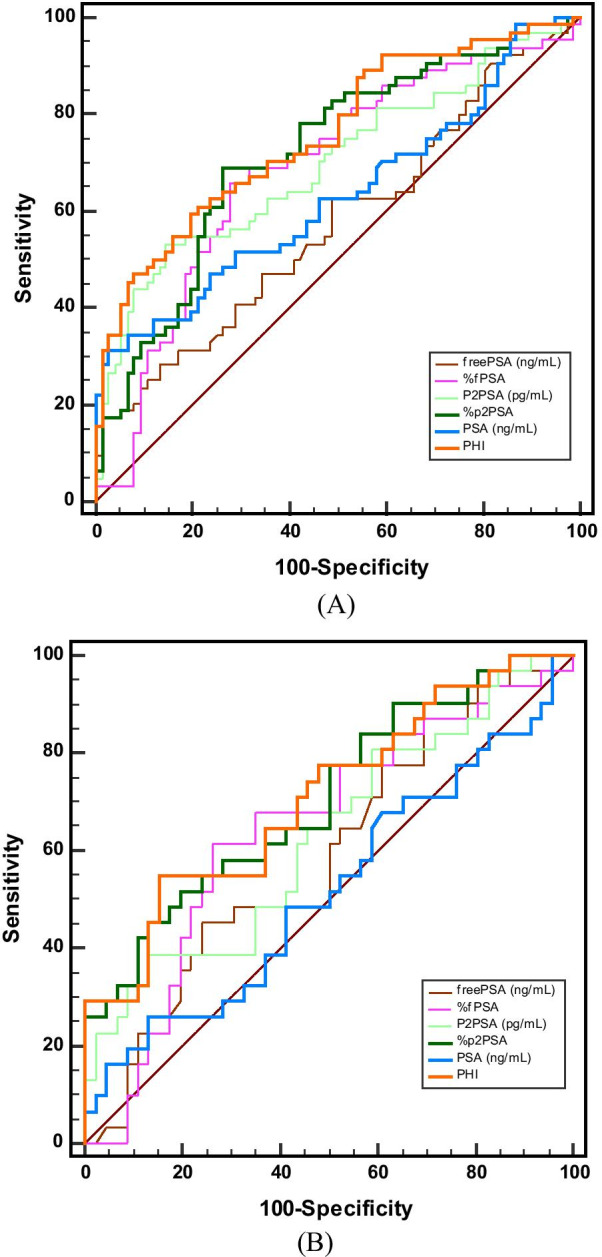
ROC curves depicting the accuracy of individual predictors of prostate cancer. **A** ROC curves in all subjects. **B** ROC curves in subjects with tPSA 4–10 ng/mL. PSA, prostate-specific antigen; fPSA, free PSA; p2PSA, [-2]proPSA; PHI, prostate health index; ROC, receiver operating characteristic; tPSA, total PSA

In 140 patients, the AUC for tPSA, fPSA, %fPSA, p2PSA, %p2PSA, and PHI were 0.63, 0.57, 0.69, 0.69, 0.72, and 0.76, respectively (Fig. 1)

Additionally, we analysed the predictive value of individual markers for predicting the probability of PCa for different age groups. Using %p2PSA and PHI had similar predictive values for different age groups, although there were some differences in the predictive value among markers for different age groups (Additional file 1: Table S1).

Using tPSA as a standard, the AUC was significantly greater in the PHI group (*p* = 0.005). Both %p2PSA and PHI were strong independent predictive markers (*p* <  0.001, *p* <  0.001) and significantly increased the predictive accuracy of a base multivariable model, including age, tPSA, fPSA and %fPSA, using multivariate logistic regression analysis (Table 4).

**Table 4:** Logistic regression analyses predicting the probability of prostate cancer

Predictors	AUC of individual predictor variable (95% CI); *P* value*	Bivariate analysis OR(95%CI); *P* value	Multivariate analysis		
			Base model ** OR (95%CI); *P* value	Base model + %p2PSA OR (95%CI); *P* value	Base model + PHI OR (95%CI); *P* value
Age	0.66 (0.57–0.74) *0.470*	1.077 (1.03–1.13); *0.002*	**1.063** **(1.01–1.12);*0.014***	**1.070** **(1.02–1.13);*0.011***	**1.068** **(1.01–1.13);*0.013***
tPSA, ng/mL	0.63 (0.54 − 0.71) -	1.100 (1.04–1.16); *0.001*	1.064 (0.96–1.18); *0.252*	1.049 (0.95 − 1.16); *0.335*	1.013 (0.93–1.10); *0.761*
fPSA	0.57 (0.48–0.65) *0.117*	1.543 (1.06–2.25); *0.024*	1.036 (0.51–2.10); *0.921*	1.012 (0.52–1.97); *0.971*	0.886 (0.48–1.65); *0.701*
%fPSA	0.69 (0.61–0.77) *0.203*	0.927 (0.88–0.98); *0.004*	0.943 (0.87–1.03); *0.181*	0.947 (0.87–1.03); *0.193*	0.959 (0.89–1.04); *0.294*
p2PSA, pg/mL	0.69 (0.60–0.76) *0.157*	1.021 (1.01–1.04); *0.001*	–	–	–
%p2PSA	0.72 (0.63–0.78) *0.109*	1.708 (1.31–2.22); *< 0.001*	–	**1.550** **(1.18–2.04);*0.002***	–
PHI	**0.76** **(0.67–0.82)*0.005***	1.017 (1.01–1.02); *< 0.001*	–	–	**1.015** **(1.01–1.02);*0.002***
AUC of multivariate models (95% CI);	–	–	**0.736** **(0.66–0.81)** ***< 0.001***	**0.793** **(0.72–0.86)** ***< 0.001***	**0.796** **(0.72–0.86)** ***< 0.001***
Gain in predictive accuracy (95% CI); *P* value	–	–	**–**	**0.056** **(− 0.0–0.11);** ***0.058***	**0.060** **(0.00–0.12);** ***0.037***

AUC, area under the receiver operating characteristic curve; PSA, prostate-specific antigen; fPSA, free PSA; p2PSA, [-2]proPSA; PHI, prostate health index; tPSA, total PSA; OR, odds ratio; CI, confidence interval

*P value: Comparison of AUC using tPSA as standard

**Base model includes age, tPSA, fPSA and %fPSA

P value to be statiscally significant with bolditalics

Similarly, in the subgroup of patients with tPSA 4–10 ng/mL, both %p2PSA and PHI were strong independent predictors (*p* = 0.007, *p* = 0.005) and showed significantly improved predictive accuracy in addition to a base multivariable model using multivariate logistic regression analysis (Table 5).

**Table 5:** Logistic regression analyses predicting probability of prostate cancer in subjects with tPSA 4–10 ng/mL

Predictors	AUC of individual predictor variable (95% CI); P value*	Bivariate analysis OR(95%CI); *P value*	Multivariate analysis		
			Base model ** OR (95%CI); *P value*	Base model + %p2PSA OR (95% CI); *P value*	Base model + PHI OR(95%CI); *P value*
Age	0.63 (0.51–0.74) *0.235*	1.069 (1.01–1.13); *0.031*	**1.081** **(1.02–1.15);*0.013***	**1.084** **(1.01–1.16);*0.018***	**1.084** **(1.01–1.16);*0.018***
tPSA, ng/mL	0.50 (0.38–0.61) **–**	1.024 (0.76–1.38); *0.875*	1.615 (0.66–3.86); *0.281*	1.384 (0.49 − 3.88); *0.536*	1.243 (0.44–3.48); *0.679*
fPSA	0.59 (0.47–0.70) *0.418*	0.529 (0.18–1.53); *0.240*	0.038 (0.00–11.2); *0.261*	0.070 (0.00–53.9); *0.433*	0.071 (0.00–56.5); *0.437*
%fPSA	0.63 (0.52–0.74) *0.090*	0.961 (0.89–1.03); *0.284*	1.155 (0.80–1.66); *0.448*	1.108 (0.73–1.69); *0.631*	1.108 (0.73–1.69); *0.632*
p2PSA, pg/mL	0.59 (0.47–0.70) *0.286*	1.023 (1.00–1.05); *0.089*	–	–	–
%p2PSA	**0.70** **(0.59–0.81)*0.038***	1.020 (1.00–1.04); *0.007*	–	**1.022** **(1.01–1.04);*0.007***	–
PHI	**0.70** **(0.59–0.81)*0.020***	1.743 (1.19–2.56); *0.005*	–	–	**1.760** **(1.17–2.64);*0.006***
AUC of multivariate models (95% CI);	–	–	**0.682** **(0.57–0.78)** ***0.003***	**0.784** **(0.68–0.87)** ***< 0.001***	**0.787** **(0.68–0.87)** ***< 0.001***
Gain in predictive accuracy (95% CI); *P* value	–	–	**–**	**0.102** **(− 0.00–0.21);** ***0.054***	**0.104** **(0.00–0.21);** ***0.048***

AUC, area under the receiver operating characteristic curve; PSA, prostate-specific antigen; fPSA, free PSA; p2PSA, [-2]proPSA; PHI, prostate health index; tPSA, total PSA, OR, odds ratio; CI, confidence interval

*P value: Comparison of AUC using tPSA as standard

**Base model includes age, tPSA, fPSA and %fPSA

P value to be statiscally significant with bolditalics

In Table 6, the results of univariate and multivariable logistic regression analyses identifying the predictors of PCa with a Gleason score ≥ 7 are presented. %p2PSA and PHI significantly improved the predictive accuracy of a base multivariable model. (*p* = 0.002, *p* = 0.001).

**Table 6:** Logistic regression analyses predicting the probability of Gleason score ≥ 7 disease

Predictors	AUC of individual predictor variable (95% CI);) P value*	Bivariate analysis OR(95%CI); *P value*	Multivariate analysis		
			Base model ** OR(95% CI); *P value*	Base model + %p2PSA OR (95% CI); *P value*	Base model + PHI OR(95%CI); *P value*
Age	0.55 (0.42–0.67) *0.508*	1.028 (0.95–1.12); *0.513*	1.005 (0.92–1.09); *0.913*	1.050 (0.95–1.16); *0.357*	1.028 (0.93–1.13); *0.577*
tPSA, ng/mL	0.62 (0.48–0.77) **–**	1.064 (0.99–1.14); *0.086*	1.031 (0.92–1.16); *0.602*	1.016 (0.940–1.098); *0.688*	0.974 (0.90–1.05); *0.498*
fPSA	0.58 (0.44–0.72) *0.358*	1.818 (0.84–3.94); *0.129*	1.399 (0.39–5.04); *0.608*	0.943 (0.36–2.46); *0.904*	0.727 (0.28–1.88); *0.511*
%fPSA	0.41 (0.26–0.57) *0.302*	0.973 (0.91–1.04); *0.440*	0.977 (0.88–1.09); *0.675*	0.978 (0.86–1.11); *0.731*	1.010 (0.90–1.13); *0.869*
p2PSA, pg/mL	**0.79** **(0.68–0.91)*0.004***	1.047 (1.01–1.09); *0.017*	–	–	–
%p2PSA	**0.86** **(0.75–0.93)*0.001***	1.020 (1.01–1.04); *0.007*	–	**1.029** **(1.01–1.05);*0.015***	–
PHI	**0.87** **(0.76–0.94)** ***< 0.001***	3.887 (1.73–8.74); *0.001*	–	–	**3.833** **(1.56–9.40);*0.003***
AUC of multivariate models (95% CI)	–	–	0.629 (0.50–0.75) *0.086*	**0.861** **(0.75–0.94)** ***< 0.001***	**0.886** **(0.78–0.95)** ***< 0.001***
Gain in predictive accuracy (95% CI); *P* value	–	–	**–**	**0.232** **(0.08–0.38);** ***0.002***	**0.257** **(0.11–0.40);** ***0.001***

AUC, area under the receiver operating characteristic curve; PSA, prostate-specific antigen; fPSA, free PSA; p2PSA, [-2]proPSA; PHI, prostate health index; tPSA, total PSA; OR, odds ratio; CI, confidence interval

*P value: Comparison of AUC using tPSA as standard

**Base model includes age, tPSA, fPSA and %fPSA

P value to be statiscally significant with bolditalics

Table 7 shows the number of patients in whom unnecessary biopsies could be avoided and the number and pathologic characteristics of cancers that would be missed using %fPSA, p2PSA, %p2PSA, and PHI at a cutoff level with 90% sensitivity in the subgroup with tPSA 4–10 ng/mL and the entire population, respectively.

**Table 7:** Cut-off of 90% sensitivity of the markers in subjects with tPSA 4–10 ng/mL

Marker	Cut-off at 90% sensitivity	% Specificity	Unnecessary biopsy avoided	Missed cancer			
				Total	Missed GS 6	Missed GS 7	Missed GS ≥ 8
*Total PSA 4–10 ng/mL *							
%fPSA	≤ 7.86	9%	4 (5.2%)	3 (10.3%)	0 (0%)	1 (3.5%)	2 (6.8%)
p2PSA (pg/mL)	≥ 8.08	17%	7 (9.1%)	3 (10.3%)	2 (6.8%)	0 (0%)	1 (3.5%)
% p2PSA	**≥ 1.20**	**27%**	**12** **(15.5%)**	**3 (10.3%)**	**3 (10.3%)**	**0** **(0%)**	**0** **(0%)**
PHI	**≥ 26.33**	**25%**	**9** **(11.7%)**	**2** **(6.8%)**	**2** **(6.8%)**	**0** **(0%)**	**0** **(0%)**
*All study subjects *							
%fPSA	≤ 6.42	7%	6 (4.3%)	7 (11.1%)	0 (0%)	2 (3.2%)	5 (7.9%)
p2PSA (pg/mL)	≥ 8.07	20%	16 (11.4%)	7 (11.1%)	4 (6.3%)	2 (3.2%)	1 (1.6%)
% p2PSA	≥ 1.22	31%	24 (17.1%)	7 (11.1%)	7 (11.1%)	0 (0%)	0 (0%)
PHI	**≥ 33.40**	40%	**32** **(22.9%)**	**5** **(7.9%)**	**5** **(7.9%)**	**0** **(0%)**	**0** **(0%)**

PSA, prostate-specific antigen; fPSA, free PSA; p2PSA, [-2]proPSA; PHI, prostate health index

Bold types to represent clinical significance could avoid unnessasary biopsies for %p2SPA and PHI

At a cutoff PHI value of 33.4, 22.9% (32/140) of patients could have avoided unnecessary biopsies without missing any significant aggressive cancers (GS ≥ 7). In the subgroup with tPSA 4–10 ng/mL, the use of %p2PSA (12/77, 15.5%) and PHI (9/77, 11.7%) could have avoided unnecessary biopsies without missing patients with aggressive cancers.

**Fig. 2:**
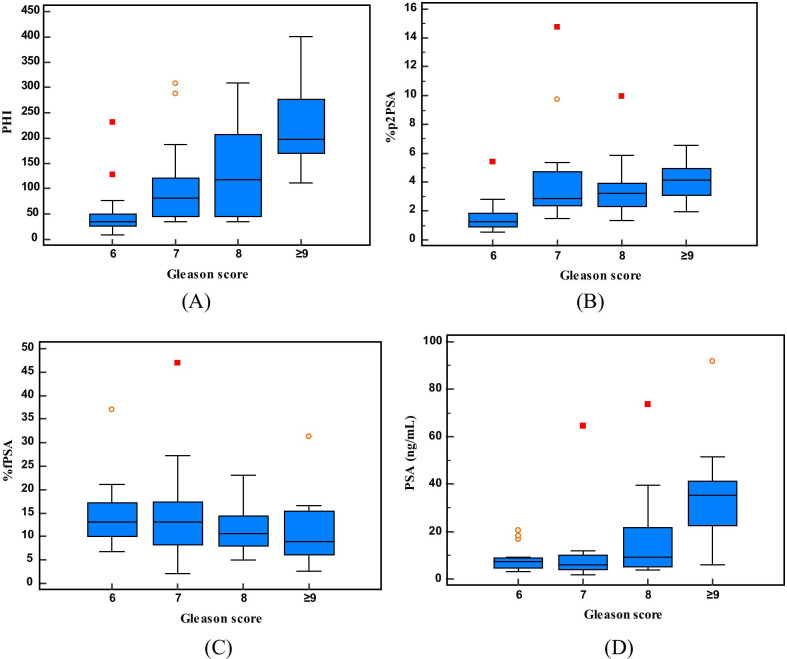
Box plots comparing PHI (**A**), %p2PSA (**B**), %fPSA (**C**) and tPSA (**D**) in relation to a biopsy Gleason score. PSA, prostate-specific antigen; fPSA, free PSA; p2PSA, [-2]proPSA; PHI, prostate health index; tPSA, total PSA

Patients with aggressive cancers had higher PHI scores. Compared to the other markers, the median values of the PHI score showed a more obvious stepwise increase along the Gleason score (Fig. 2).

## Discussion

This study evaluated the usefulness of %p2PSA and PHI in 140 subjects, and our findings support previous results regarding both biomarkers. %p2PSA and PHI showed higher predictive performance in the detection of PCa compared to standard reference methods, and they were better able to distinguish aggressive (GS ≥ 7) from clinically indolent PCa. Thus, their use could avoid unnecessary biopsies without missing clinically significant cancers.

Currently, PSA is widely used for PCa screening, but the limitations of PSA as a biomarker for PCa detection have been well demonstrated. It is necessary to distinguish PCa from benign prostatic disease and to clarify the aggressiveness of cancers, but PSA cannot completely predict the presence and biological behaviour of PCa [13]. The early detection of PCa using PSA results in a large number of negative biopsies and a high proportion of patients diagnosed with clinically low aggressive tumours (over-diagnosis) followed by unnecessary treatment (over-treatment) and morbidity related to complications [14, 15]. Thus, a more specific biomarker that could increase predictive accuracy and risk stratification properties is needed to identify patients who may have PCa and reduce morbidity due to unnecessary diagnosis and treatment.

The usefulness of %p2PSA and PHI in the detection of PCa has been studied extensively in recent years. The biomarkers improve the specificity of tPSA for PCa detection and are associated with a more aggressive state of disease [5, 13].

Catalona et al. demonstrated that high PHI levels were associated with an increased detection rate of PCa in subjects with a tPSA level between 2 and 10 ng/ml in a prospective multi-institutional study [16]. Jansen et al. showed that PHI showed significantly superior performance compared to PSA and %fPSA for PCa prediction, and the involvement of p2PSA in a base multivariable model significantly improved the predictive value and specificity of PCa [17].

Several studies have also validated the usefulness of PHI in Asian countries. Chiu et al., in their prospective study, showed that PHI improved the diagnostic accuracy compared with PSA-based predictive models in 569 subjects with PSA levels between 4 and 10 ng/mL in Hong Kong [18]. In a multicentre study in Shanghai, Na et al. demonstrated the superior diagnostic accuracy of PHI compared to tPSA both in subjects with a PSA level between 2.1 and 10 ng/mL and in those with a PSA level > 10 ng/mL [19].

One meta-analysis showed that %p2PSA and PHI consistently improved diagnostic performance compared to tPSA and %fPSA in detecting PCa and could reduce unnecessary biopsies [20]. In addition, a European prospective study showed that PHI showed improved predictive performance for GS ≥ 7 PCa [21]. The 2016 guidelines of the European Association of Urology suggested that PHI could be considered as an additional diagnostic method for patients with PSA levels of 2–10 ng/mL and a negative DRE [22].

In the current study, the addition of %p2PSA or PHI to a predictive base multivariate regression model significantly increased its predictive accuracy. Moreover, %p2PSA and PHI were associated with aggressiveness of PCa and could improve the predictive performance of the base model for detecting GS ≥ 7 PCa. The proportion of aggressive cancer was mostly associated with the PHI level among these markers. At a cutoff PHI value of 33.4, 22.9% (32/140) of biopsies could have been avoided without missing any significant aggressive cancers (GS ≥ 7). In the same context, at a cutoff PHI value of 26.3, 11.7% (9/77) of biopsies would have been avoided without missing any significant aggressive cancers (GS ≥ 7) in subjects with a tPSA 4–10 ng/mL. Similarly, another study demonstrated that 15.5–45.2% of their group could have avoided unnecessary biopsies at a cut-off of PHI score 25–32, although they would have missed 1.1–3.8% of significant aggressive cancers [23, 24].

The European population had a fourfold higher incidence of PCa than the Asian population, while age and PSA level showed a tendency to be higher among Asians in a previous study [25]. Korean men also have a lower incidence of PCa compared to the Western population, but PCa in the Korean population shows worse characteristics of the disease compared to Western men [3, 4]. Most of the previously reported data regarding %p2PSA and PHI have been collected mainly in Western groups; therefore, it is necessary to verify the usefulness of these biomarkers in Korean groups. Kim et al. evaluated the clinical predictive value of %p2PSA and PHI in Korean men [26]. Similar to previous studies, they suggested that the diagnostic accuracy of PHI was better than that of tPSA in the Korean population.

Recently, multiparametric magnetic resonance imaging (MRI) has improved the detection rate of potentially significant PCa [27], but it generally requires higher costs and radiological expertise. It has been reported that MRI and PHI are complementary to each other for detecting significant PCa [28]. PHI is a blood test that can be performed simply and is ordered by general practitioners, and there is no need for radiologic interpretation. In the future, as the cost of a blood test will probably decrease, PHI will be widely used as a screening tool for PCa.

This study has several limitations. First, it was performed in a single tertiary centre and had a relatively small sample size. Second, not all patients in our study underwent radical prostatectomy; therefore, we could not inspect the occurrence of Gleason upgrading after prostatectomy. In addition, we did not inspect the percentage of tumour involvement in each biopsy core and tumour size. Finally, we did not use multiparametric MRI. MRI could help guide more accurate localisation for biopsy and increase the performance for detecting significant PCa.

## Conclusions

Our findings suggest that the diagnostic performance of %p2PSA and PHI to predict the presence and aggressiveness of PCa was superior to that of PSA and %fPSA in the Korean population. Using PHI, a high proportion of unnecessary biopsies could be avoided. Further research is needed to support these results.

## Supplementary Information

Additional file 1: Table S1. Predictive value of individual markers predicting the probability of prostate cancer for different age groups.

## Data Availability

The datasets used and/or analysed during the current study are available from the corresponding author on reasonable request.

## Abbreviations

AUC: Area under the curve
fPSA: Free PSA
GS: Gleason score
p2PSA: [− 2]proPSA
PCa: Prostate cancer
PHI: Prostate health index
PSA: Prostate-specific antigen
ROC: Receiver operating characteristic
tPSA: Total PSA
