# Supplementary material for: Usefulness of the prostate health index in predicting the presence and aggressiveness of prostate cancer among Korean men: a prospective observational study
Source: BMC Urol. 2021 Sep 16;21:131. doi: 10.1186/s12894-021-00897-2 (PMC8447777; doi:10.1186/s12894-021-00897-2)
Supplement: Supplementary file 1 — Predictive value of individual markers predicting the probability of prostate cancer for different age groups. [file 12894_2021_897_MOESM1_ESM.docx]

**Additional file 1: Table S1. Predictive value of individual markers predicting the probability of prostate cancer for different age groups**

|  | **<60** (N,15) | **60-69** (N,60) | **70-79** (N,51) | **≥80** (N,14) |
| --- | --- | --- | --- | --- |
| Predictors | AUC (95% CI); *P value* | AUC (95% CI); *P value* | AUC (95% CI); *P value* | AUC (95% CI); *P value* |
| **tPSA, ng/mL** | 0.760  (0.48-0.94)  *0.086* | 0.508  (0.38-0.64)  *0.923* | **0.702**  **(0.56-0.82)**  ***0.006*** | 0.644  (0.35-0.87)  *0.367* |
| **fPSA** | 0.760  (0.48-0.94)  *0.063* | 0.624  (0.39-0.75)  *0.101* | 0.617  (0.47-0.75)  *0.141* | 0.600  (0.31-0.84)  *0.575* |
| **%fPSA** | 0.560  (0.29-0.81)  *0.734* | **0.650**  **(0.48-0.76)**  ***0.049*** | **0.660**  **(0.50-0.78)**  ***0.043*** | 0.644  (0.35-0.87)  *0.380* |
| **p2PSA, pg/mL** | **0.940**  **(0.69-0.99)**  ***<0.001*** | 0.596  (0.46-0.72)  *0.272* | **0.670**  **(0.52-0.80)**  ***0.025*** | **0.867**  **(0.58-0.99)**  ***0.001*** |
| **%p2PSA** | **0.920**  **(0.66-0.99)**  ***<0.001*** | **0.714**  **(0.58-0.82)**  ***0.005*** | **0.678**  **(0.53-0.80)**  ***0.021*** | **0.844**  **(0.56-0.98)**  ***0.029*** |
| **PHI** | **0.920**  **(0.66-0.99)**  ***<0.001*** | **0.715**  **(0.58-0.82)**  ***0.004*** | **0.730**  **(0.59-0.85)**  ***0.001*** | **0.911**  **(0.64-0.99)**  ***<0.001*** |

AUC, area under the receiver operating characteristic curve; PSA, prostate-specific antigen; fPSA, free PSA; p2PSA, [-2]proPSA; PHI, prostate health index; tPSA, total PSA, CI = confidence interval
